# Early implantation of a transjugular intrahepatic portosystemic shunt (TIPS) in patients with liver cirrhosis and ascites (eTIPS): a multicentre, randomised controlled trial

**DOI:** 10.1186/s13063-025-09038-8

**Published:** 2025-10-02

**Authors:** Dominik Bettinger, Marco Janoschke, Carolin Jenkner, Margit Kaufmann, Julia van Gessel, Hans-Heinrich Otter, Michael Schultheiss, Robert Thimme

**Affiliations:** 1 Department of Medicine II, Faculty of Medicine, Medical Center University of Freiburg, University of Freiburg, Hugstetter Str. 55, Freiburg, 79106 Germany; 2 Clinical Trials Unit, University Medical Center, University of Freiburg, Freiburg, Germany; 3 Faculty of Medicine, Berta-Ottenstein-Programme, University of Freiburg, Freiburg, Germany

**Keywords:** Cirrhosis, Ascites, Transjugular intrahepatic portosystemic shunt, Transplantation, Survival

## Abstract

**Background:**

Portal hypertension is a major complication in patients with liver cirrhosis, leading to severe outcomes such as variceal bleeding and ascites. Transjugular intrahepatic portosystemic shunt (TIPS) has emerged as an effective interventional treatment of recurrent ascites and variceal bleeding. However, up to 30% of patients with recurrent ascites show TIPS refractory ascites, and prior data have shown that the frequency of paracenteses before TIPS implantation predicts ascites clearance indicating that TIPS implantation may be too late in some patients. Especially, patients with grade 2 ascites and a MELD score ≥ 15, or grade 3 ascites irrespective of MELD score at first decompensation with ascites face a high risk of further decompensation and mortality. Therefore, these patients may benefit from early TIPS implantation in order to improve post-TIPS mortality. We hypothesise that early TIPS implantation in these selected patients at the time of the first decompensation may improve transplantation-free survival compared to standard medical treatment (SMT).

**Methods:**

The eTIPS study is a prospective, randomised, open, multicenter interventional, superiority trial. Patients will be randomised 1:1 in the intervention group with TIPS implantation and in the SMT group. The primary endpoint is transplantation-free survival. Secondary endpoints include the time to ascites with need for paracentesis and quality of life assessed six and 12 months after randomisation.

**Discussion:**

Expanding the concept of early TIPS implantation to ascites management may offer significant survival benefits and may significantly change the treatment algorithm of patients with ascites.

**Trial registration:**

German Registry for Clinical Studies DRKS00034545. Registered on 20/02/2025.

Clinical trials NCT06576934. Registered on 04/12/2024.

## Administrative information

Note: the numbers in curly brackets in this protocol refer to SPIRIT checklist item numbers. The order of the items has been modified to group similar items (see http://www.equator-network.org/reporting-guidelines/spirit-2013-statement-defining-standard-protocol-items-for-clinical-trials/).

**Table:** 

Title {1}	Early implantation of a transjugular intrahepatic portosystemic shunt (TIPS) in patients with liver cirrhosis and ascites: a multicentre, randomised controlled trial
Trial registration {2a and 2b}	NCT06576934 DRKS00034545
Protocol version {3}	version 1.3., 03.07.2025
Funding {4}	This clinical study is funded by the German Research Foundation (Deutsche Forschungsgemeinschaft, DFG). GZ: BE 7734/2–1 Project number: 529465923
Author details {5a}	Prof. Dr. Dominik Bettinger Department of Medicine II, Medical Center—University of Freiburg, Hugstetter Str. 55, D-79106 Freiburg, Germany dominik.bettinger@uniklinik-freiburg.de Dr. Marco Janoschke Medical Center – University of Freiburg, Clinical Trials Unit, Elsässer Str. 2, 79,110 Freiburg, Germany marco.janoschke@uniklinik-freiburg.de Dr. Carolin Jenkner Medical Center – University of Freiburg, Clinical Trials Unit, Elsässer Str. 2, 79,110 Freiburg, Germany carolin.jenkner@uniklinik-freiburg.de Dr. Margit Kaufmann Medical Center – University of Freiburg, Clinical Trials Unit, Elsässer Str. 2, 79,110 Freiburg, Germany margit.kaufmann@uniklinik-freiburg.de Julia van Gessel Medical Center – University of Freiburg, Clinical Trials Unit, Elsässer Str. 2, 79,110 Freiburg, Germany julia.vangesssel@uniklinik-freiburg.de Hans-Heinrich Otter Medical Center – University of Freiburg, Clinical Trials Unit, Elsässer Str. 2, 79,110 Freiburg, Germany hans-heinrich.otter@uniklinik-freiburg.de PD Dr. Michael Schultheiss Department of Medicine II, Medical Center—University of Freiburg, Hugstetter Str. 55, D-79106 Freiburg, Germany michael.schultheiss@uniklinik-freiburg.de Prof. Dr. Robert Thimme Department of Medicine II, Medical Center—University of Freiburg, Hugstetter Str. 55, D-79106 Freiburg, Germany robert.thimme@uniklinik-freiburg.de
Name and contact information for the trial sponsor {5b}	Medical Center—University of Freiburg represented by the Chief Medical Officer (CMO, *Leitende Ärztliche Direktion)* and the Chief Financial Officer (CFO, *Kaufmännische Direktion*), Breisacher Str. 153, 79,110 Freiburg, Germany
Role of sponsor {5c}	The Medical Center- University of Freiburg is the study sponsor. The sponsor does not have a role in study design, collection, management and analysis of data, interpretation of data and writing of the report or the decision regading publication.

## Introduction

### Background and rationale {6a}

Portal hypertension is a hallmark of liver cirrhosis leading to oesophageal and gastric varices, variceal bleeding and ascites [1–3]. These complications may trigger further complications such as hepatorenal syndrome and spontaneous bacterial peritonitis, and they are associated with significant morbidity and mortality [2, 4]. Implantation of a transjugular intrahepatic portosystemic shunt (TIPS) is a safe and effective interventional treatment for portal hypertension, and TIPS implantation has emerged as a major component within the treatment algorithm for portal hypertension [5, 6]. The most common indications for TIPS implantation are secondary prophylaxis of variceal bleeding and recurrent or treatment refractory ascites [5]. According to recent evidence, early TIPS (i.e. pre-emptive TIPS) within the first 72 h following the bleeding event in patients with acute variceal bleeding and a high risk of re-bleeding (CHILD–Pugh C < 14 or CHILD–Pugh B > 7 with active bleeding in index endoscopy) is associated with improved survival [7]. Even patients with acute-on-chronic liver failure (ACLF) after variceal bleeding show a survival benefit after pre-emptive TIPS implantation [8]. Therefore, the concept of early TIPS implantation after variceal bleeding is recommended by current guidelines [1, 7].

In contrast, patients with ascites are allocated to TIPS implantation at later stages of disease (treatment refractory or recurrent ascites) when diuretic treatment and large-volume paracenteses (LVP) do not lead to sufficient ascites control [1]. The clinical benefit of TIPS implantation has been confirmed in several randomised controlled trials, although these studies showed very heterogeneous results with respect to improvement of survival [1, 9–11]. In the majority of these studies, uncovered stents were used, but today, the use of covered stents is exclusively recommended due to their superior patency. Only in the study by Bureau et al. covered stents were used, and thus this study may be considered representative for everyday clinical practice [12]. A clear survival benefit for patients allocated to TIPS implantation was shown, and the prevalence of post-TIPS hepatic encephalopathy (HE) was not increased in these patients. Patients who received TIPS implantation due to recurrent or refractory ascites show ascites clearance without the need for further paracenteses in only 51% to 68% [1, 11]. In contrast, the study by Bureau et al. reported a significantly improved treatment response, and treatment failure occurred in only 29% of patients treated with TIPS implantation [12]. Improved response to treatment and increased survival may be explained by meticulous patient selection, as only patients with recurrent ascites were included in this study. These patients had no more than six paracenteses within the last three months, and patients were allocated to TIPS implantation after a mean of 4.5 paracenteses indicating that these patients did not fulfil the definition of refractory ascites [12]. These facts highlight the rationale for early allocation to TIPS implantation.

The benefit of timely TIPS implantation (i.e. following recurrent ascites instead of refractory ascites) was supported by the study from Piecha et al. [13]. In this study, a higher frequency of paracenteses as a surrogate marker for the duration of decompensation and a high serum creatinine were associated with a reduced clearance of ascites after TIPS implantation [13]. These data support to expand the concept of “early TIPS implantation” to patients with ascites. Balcar et al. analysed the clinical course of patients with ascites as the first decompensating event. They were able to show that patients with grade 2 ascites and a MELD score ≥ 15 as well as patients with grade 3 ascites irrespective of the MELD score had a high risk of further decompensation and increased risk for death [14]. We hypothesise that these patients may benefit from early TIPS implantation. This highly relevant clinical question has not yet been addressed in a prospective randomised controlled trial. Therefore, we aim to analyse the concept of “early TIPS implantation” in these selected patients with liver cirrhosis and ascites as the first single decompensating event.

### Objectives {7}

The primary objective of this study is to assess a survival benefit in patients with early allocation to TIPS implantation in comparison to patients with SMT. The secondary objectives aim to assess the efficacy of TIPS implantation on the course of liver disease and quality of life (QOL).

### Trial design {8}

The eTIPS study is a prospective, randomised, open, multicentre interventional, superiority trial. Patients will be randomised 1:1 in the intervention group with TIPS implantation and in the SMT group. Blinding is not possible because of the interventional character of TIPS implantation.

**Fig. 1:**
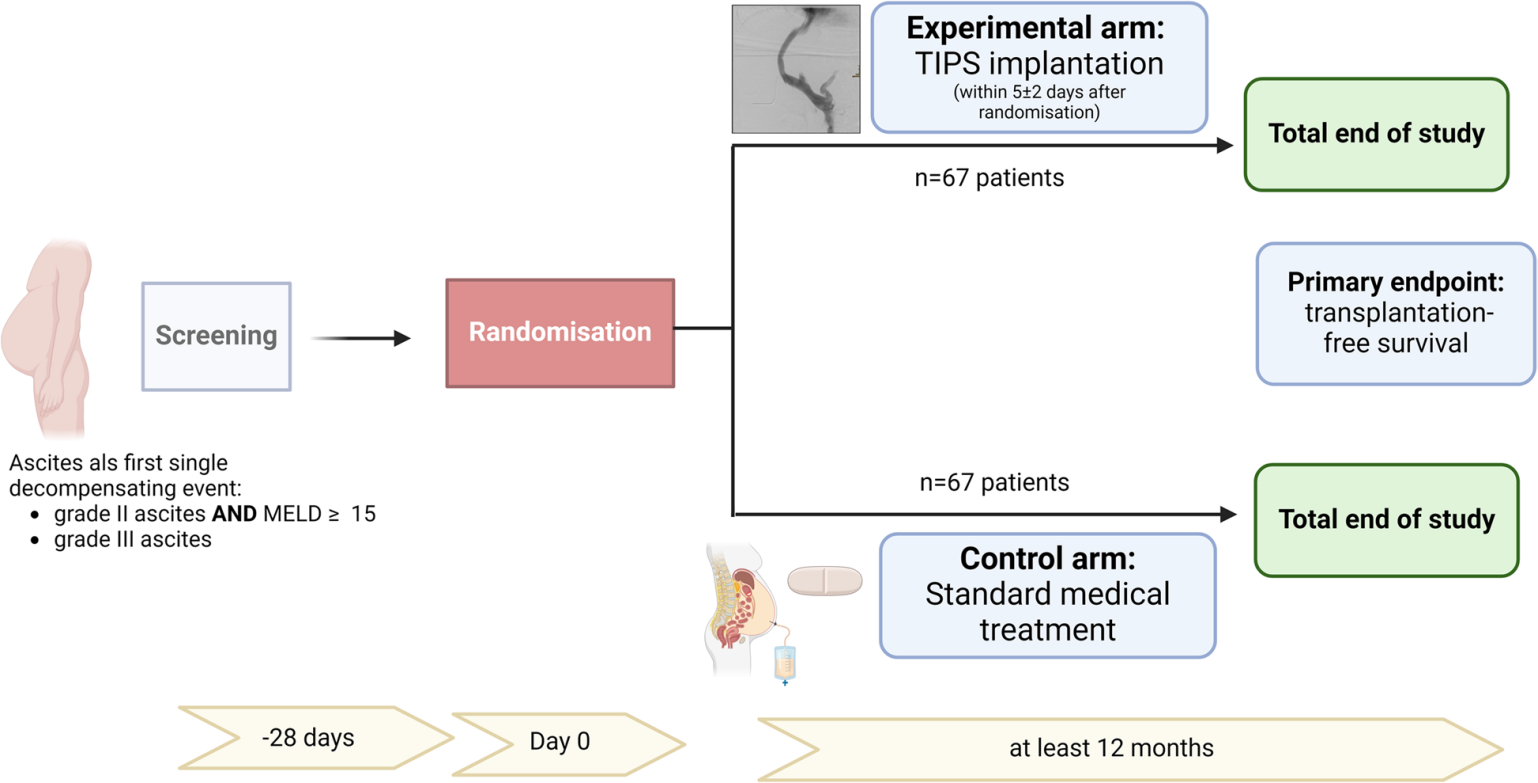
Trial design of the eTIPS study (created with biorender.com)

We hypothesise that early TIPS implantation in cirrhotic patients with first decompensation with ascites leads to improved transplantation-free survival compared to SMT. The trial design is shown in Fig. 1.

## Methods: participants, interventions and outcomes

### Study setting {9}

This is a multicentre trial that will be performed in approximately 10 tertiary academic centres in Germany that are highly experienced in the management of patients with advanced chronic liver disease, portal hypertension and TIPS implantation. List of participating sites can be obtained from continuously updated entries found in the registry on clinicaltrials.gov and in the German registry for Clinical Trials (DRKS). If necessary, additional qualified centres can be included during the study period. All participating centres must provide a multidisciplinary team including hepatologists, interventional radiologists (only in case TIPS implantations are performed by them), intensive care and general surgery with expertise in liver surgery and liver transplantation. The TIPS procedure should be done by an interventional radiologist or an interventional hepatologist who has experience in this procedure (performance of at least 5 TIPS intervention under supervision and at least 15 TIPS procedures without supervision). This qualification will be asked during the site selection visit by self-assessment. If there are personal changes during the study period, the sites are responsible for providing these data for the persons involved in TIPS implantation.

### Eligibility criteria {10}

The eligibility criteria are summarised in Table 1. No gender ratio has been stipulated in this trial as the results of the preclinical and clinical studies did not indicate any gender effect of the trial treatment in terms of efficacy and safety.

**Table 1:** Eligibility criteria

Inclusion criteria	Exclusion criteria
Patients eligible for inclusion in this trial must meet *all* of the following criteria:	Patients eligible for this trial must *not* meet any of the following criteria:
1. Patients ≥ 18 years and < 80 years. (Patients > 80 years independent of their physical constitution are excluded as they show higher complication rates and mortality after TIPS implantation) 2. Liver cirrhosis as documented by previous liver biopsy or by a combination of typical clinical, biochemical and sonographic features 3. Ascites as the first single decompensating event with grade 2 ascites and MELD ≥ 15 or grade 3 ascites [1] 4. Ability to understand the nature of the trial and the trial related procedures and to comply with them	1. Treatment refractory or recurrent ascites at the time of study inclusion 2. Patients with concomitant variceal bleeding fulfilling the criteria for pre-emptive TIPS implantation (Child–Pugh class C < 14 points or Child–Pugh class B > 7 with active bleeding at initial endoscopy or hepatic venous pressure gradient [HVPG] > 20 mmHg at the time of bleeding) 3. Budd-Chiari syndrome 4. Portal vein thrombosis (PVT) 5. Spontaneous bacterial peritonitis (SBP) 6. Uncontrolled systemic infection (defined as an increase of > 20% if inflammatory parameters [C-reactive protein, procalcitonin, leukocytes] and/or sepsis as a reason for development of ascites 7. Cardiac cirrhosis (defined as the development of liver cirrhosis in a patient with cardiac heart failure due to primary cardiac disease) 8. Clinical significant cardiac disease (NYHA ≥ II) 9. Untreated valvular heart disease: middle to high-grade valve stenosis or insufficiency (applies to mitral, tricuspid, aortic and pulmonary valves) 10. Diastolic dysfunction grade III, stated by transthoracic echocardiogram (TTE) 11. Reduced left ventricular ejection fraction ≤ 50% 12. Pulmonary hypertension (mean pulmonary arterial pressure > 45 mmHg) 13. Bilirubin > 3 mg/dl 14. Obstructive cholestasis 15. Hepatorenal syndrome type AKI 16. Acute on chronic liver failure 17. Benign liver tumor e.g. cysts within the potential puncture tract 18. Patient after liver transplantation 19. Prior TIPS implantation 20. Ongoing and/or recurrent hepatic encephalopathy (grade ≥ II) 21. Active tumor disease including hepatocellular carcinoma defined as need for chemotherapy, radiation therapy, interventional or surgical treatment 22. New onset of antiviral treatment for chronic hepatitis B virus (HBV) infection within the last 3 months 23. Untreated chronic hepatitis C virus (HCV) infection 24. Life expectancy < 1 year 25. Pregnant or breastfeeding women 26. Patients without legal capacity who are unable to understand the nature, significance and consequences of the study or who are judged as non-compliant (e.g. due to active drug abuse, severe alcohol abuse) 27. Simultaneous participation in other interventional trials which could interfere with this trial; simultaneous participation in registry and diagnostic trials is allowed 28. Person who is in a relationship of dependence/employment with the sponsor or the investigator

#### Who will take informed consent? {26a}

Cirrhotic patients with ascites as the first single decompensating event with grade 2 ascites and MELD score ≥ 15 **or** grade 3 ascites will be screened for eligibility. If a patient appears to be eligible for the trial, the investigator will inform the patient about the trial and ask the patient for his/her written consent. It is imperative that written consent is obtained prior to any trial-specific procedures. Importantly, the screening examinations are performed during routine clinical management and are not exclusively related to the study participation so that they may be performed prior to written informed consent.

#### Additional consent provisions for collection and use of participant data and biological specimens {26b}

Blood and urine samples are collected during the study period at pre-defined timepoints. No additional biological specimens will be sampled.

## Interventions

### Explanation for the choice of comparators {6b}

Currently, TIPS implantation is recommended for patients with liver cirrhosis and refractory or recurrent ascites [1, 7]. However, response to treatment (ascites clearance) is often reduced in these patients with advanced liver disease and long-standing decompensation with ascites [13, 15]. Therefore, we hypothesise that early TIPS implantation in selected patients with ascites as the first decompensating event (grade 2 ascites and MELD ≥ 15 or grade 3 ascites) will lead to a higher rate of ascites clearance and improved transplantation-free survival. Therefore, patients with grade 2 ascites and MELD score ≥ 15 or grade 3 ascites as the first single decompensating event will be randomised in an early TIPS group in addition to standard of care and in a SMT group. Patients in the SMT group will be treated following current evidence-based treatment guidelines [1], and therefore, the SMT group reflects the current approach in treatment of patients with ascites. All patients (also including patients in the TIPS group) are advised to adhere to moderate restriction of sodium intake (80–120 mmol/day = 4.6–6.9 g of salt) according to the current EASL guidelines [1, 16]. Treatment of ascites will be performed according to current evidence-based treatment guidelines. Diuretic treatment (using an anti-mineralocorticoid drug and/or loop diuretics) will be used by clinical judgement under regular control of renal function, sodium and potassium values. The body weight loss should not exceed 0.5 kg/day in patients without peripheral oedema and 1 kg/day in the presence of peripheral oedema. The dose of diuretics can be increased after 72 h of treatment until the maximum dose (spironolactone, 400 mg/day; furosemide, 160 mg/day; torasemid, 40 mg/day) is reached or diuretic-induced complications occur [1, 16]. Diuretic-induced complications are defined as acute kidney injury (defined according the KDIGO criteria) and/or diuretic-induced hyponatraemia (decrease of serum sodium by > 10 mmol/l or to a serum sodium < 125 mmol/l) and/or diuretic-induced hypo-or hyperkalaemia (changes in serum potassium to < 3 mmol/l or > 6 mmol/l despite appropriate measures) and/or disabling muscle cramps.

In patients with grade > 2 ascites, LVP will be performed according to current recommendations. Plasma volume expansion with albumin infusion will be performed following completion of LVP (albumin 8 g/l of ascites drained). If less than 5 l of ascites was drained, no plasma expansion is necessary [1].

The current Baveno VII guidelines make a strong recommendation for the use of non-selective beta-blockers (NSBB; preferred carvedilol or propranolol) for the prevention of decompensating events and therefore, this will be a common co-medication in the SMT group. NSBB should be stopped in case of acute kidney injury (AKI) and persistently systolic blood pressure < 90 mmHg or mean arterial pressure < 65 mmHg. After blood pressure returns to normal and AKI is resolved, NSBB can be restarted [7].

In case of development of overt hepatic encephalopathy, treatment will be started incorporating lactulose (10 ml twice or more daily as necessary) and/or rifaximin 550 mg twice daily [17].

### Intervention description {11a}

TIPS implantation has emerged as an important interventional treatment option in patients with decompensated liver cirrhosis. It leads to a significant decrease in portal vein pressure and effective control of portal hypertension related complications [5].

A high-resolution flat-panel X-ray C-arm with digital subtraction angiography is necessary. During TIPS implantation, vital signs including non-invasive blood pressure monitoring, heart rate, electrocardiogram and peripheral oxygen saturation are monitored.

TIPS implantation will be performed by experienced interventional hepatologists or radiologists (performance of at least 5 TIPS interventions under supervision and at least 15 TIPS procedures without supervision). Before TIPS implantation, LVP may be necessary to facilitate the TIPS procedure. This paracentesis will be documented on the TIPS implantation page in the eCRF and will not be considerd as a secondary endpoint.

A central vascular access for TIPS implantation is obtained by introducing a sheath (10F) in the right internal jugular vein. If the right jugular vein cannot be assessed due to thrombosis or other vascular abnormalities, the left internal jugular vein should be used. Under fluoroscopic and sonographic guidance, the TIPS needle is inserted in the hepatic vein (normally right or middle hepatic vein) and the needle is introduced through the liver parenchyma towards the (right) portal vein branch. It is recommended to use ultrasound guidance for the transhepatic puncture of the portal vein. After the portal vein has been assessed, a guidewire is introduced in the splenic vein or in the superior mesenteric vein. Portography is performed and the portosystemic gradient (PSG) is measured [7, 18, 19]. PSG is calculated as the difference between the inferior vena cava (IVC) and the pressure in the main portal vein. In addition, the pressure in the right atrium is measured. After a PSG > 10 mmHg has confirmed portal hypertension, the parenchymal tract is dilated with a 6-mm dilation balloon. An 8–10 mm controlled-expansion polytetrafluoroethylene (ePTFE)-covered stent is introduced and the stent graft is implanted. It is important that the stent is placed sufficiently within the liver vein (close to the IVC). In patients who are suitable for liver transplantation, the location of the stent is important. A too proximal stent position (stent direct to the right atrium) and too distal stent position (close to the confluence) must be avoided.

Only covered stents with controlled expansion must be used in this trial. The stent will first be dilated to 6 mm and the PSG is measured. If PSG will not show reduction to levels lower than 12 mmHg or no pressure reduction of more than 50% has been reached, the stent will be dilated to a diameter of 8, 9 or 10 mm until the reduction of PSG reaches the target pressure range (PSG < 12 mmHg or PSG reduction > 50%).

 Between each dilation, PSG must be measured and documented. In addition, systemic haemodynamic parameters (blood pressure and heart rate) at the time of PSG measurements must be added to the documentation.

If portosystemic collaterals are detected during portography, these varices or major collaterals can be embolised using metallic coils or glue according to the interventionalist’s choice, especially if these collaterals lead to a steal phenomenon with reduced inflow into the TIPS stent. TIPS implantation will normally be performed in deep sedation using centre specific sedation regimes [20]. According to local standard, general anaesthesia is permitted within the trial. Importantly, sedation has a significant impact on pressure values and therefore, a second measurement of the portal vein pressure and the pressure in the IVC and right atrium should be performed 24–72 h after TIPS implantation. If necessary, however not recommended, mild sedation with low-dose midazolam (0.02 mg/kg body weight) is permitted [7].

TIPS implantation is performed with X-ray fluoroscopy. Approximately 20 min of X-ray fluoroscopy is necessary for TIPS implantation. The second pressure measurement requires a maximum of 5 min X-ray fluoroscopy. It is within the responsibility of the interventionalist to take measures to reduce X-ray exposure (reducing the X-ray field and rate of fluoroscopy). Contrast medium is used for visualisation of vessels, and it should be reduced to a minimum. If locally available, CO_2_ angiography can be used.

### Criteria for discontinuing or modifying allocated interventions {11b}

Patients in the SMT group may be allocated to TIPS implantation according to current Baveno VII guidelines and the EASL guideline for the management of decompensated cirrhosis [1, 5, 7, 21]. TIPS implantation is indicated in the following situations:

- Massive variceal bleeding with implantation of a fully covered self-expanding metal stent (fcSEMS) for bleeding control (rescue TIPS implantation) [22].
- Variceal bleeding (esophageal or gastric varices) in patients with Child–Pugh B > 7 points and active bleeding during index gastroscopy or patients with Child–Pugh C < 14 points (pre-emptive TIPS implantation) [7].
- Recurrent esophageal bleeding in patients with Child–Pugh A or Child–Pugh B 7 points despite adequate secondary prophylaxis with beta-blockers and endoscopic band ligation (EBL) [7].
- Recurrent ascites (as defined by requirement of ≥ 3 large-volume paracenteses [removal of > 5 L of ascites] within 1 year) in stable situations can be considered for TIPS implantation. Importantly, only paracenteses in stable conditions will be considered. Stable conditions are defined as a clinical situation without concomitant infections, without acute kidney injury and/or bleeding at the time of paracentesis [1, 7].

If TIPS implantation is planned in the SMT arm, the indication should be discussed with the coordinating investigator.

### Strategies to improve adherence to interventions {11c}

The strict adherence to inclusion and exclusion criteria should improve adherence to the intervention. Further, randomisation is performed as late as possible. At each scheduled visit, the study team will reinforce the importance of treatment adherence and may help in solving upcoming problems.

### Relevant concomitant care permitted or prohibited during the trial {11d}

No medication is prohibited. Treatment with transfusions (red blood cells and platelets) and supportive care as well as intensive care treatment are permitted after the initiation of study treatment. Patients should receive treatment/medication appropriate to their clinical condition in an emergency. The patient must notify the investigational site of any new medication he/she starts taking after signature of informed consent. All concomitant medications and significant non-drug therapies (including physical therapy and blood transfusions) must be listed in the source documents and additionally documented in eCRF especially diuretic treatment, NSBB and medications for hepatic encephalopathy will be considered [23].

### Provisions for post-trial care {30}

After the patient has completed follow-up in the study, the patient will return to routine clinical practice and will receive follow-up in the outpatient clinic every 3 to 6 months according to his/her clinical condition.

All patients included in the study will have insurance in accordance with the applicable law and regulations (Insurance: Newline Group, Policy-No.: NEV081957A).

### Outcomes {12}

Table 2 summarises the objectives and the corresponding endpoints of this trial. The following estimands are defined.

**Table 2:** Objectives and endpoints in the eTIPS trial

Objective	Endpoint
**Primary**	
The primary objective of this study is to assess a survival benefit in patients with early allocation to TIPS implantation in comparison to patients with standard medical treatment (SMT)	The primary endpoint is transplantation-free survival (TFS), defined as time from randomisation to liver transplantation or death (Estimand 1)
**Secondary**	
The secondary objectives aim to assess the efficacy of TIPS implantation on the course of liver disease and quality of life (QOL)	The following outcome parameters are defined as secondary endpoints: • Time to ascites with need for paracenteses^#^ (Estimand 2) • Quality of life assessed with the SF-36 and CLDQ (chronic liver disease questionnaire) 6 and 12 months after randomisation (Estimand 3)
**Safety**	• Adverse events (AEs), and severe adverse events (SAEs), incidence of complications related to TIPS implantation (incl. technically unsuccessful TIPS implantation) • Rate of TIPS dysfunction • Rate of TIPS implantation in the SMT group

^#^Ascites with need for paracenteses will be classified as following, taking into account the *abdominal distention* caused by ascites [1]: (I): Ascites grade 1(mild): ascites is detectable only by ultrasound examination; (II): Ascites grade 2(moderate): ascites causes moderate symmetrical distension of the abdomen; (III): Ascites grade 3 (severe, large/gross ascites): it causes marked abdominal distension. If patients develop symptoms due to ascites (pain, abdominal tension, dyspnoea), paracentesis should be performed. Date of paracentesis, volume removed and albumin replacement/dose are documented for each paracentesis. Recurrent ascites according to the Baveno VII recommendations is defined as a requirement of 3 or more large-volume (greater than 5 L) paracenteses per year (only paracenteses in stable conditions without infections are considered)[7]

#### Estimand 1

The primary estimand that corresponds to the primary trial objective to show superiority of early implantation of a TIPS in patients with liver cirrhosis and ascites compared to standard medical treatment based on the primary endpoint transplantation-free survival is specified by the following 5 attributes.

*Population:* [A] The population targeted by the primary estimand will be called full analysis set (FAS). This means that the patients will be analysed in the treatment arms to which they were randomised. All randomised patients will be included in the FAS.

*Treatments***:** The randomised experimental arm (TIPS implantation) is compared to the randomised control arm (SMT) of ascites regardless of dosing modifications, treatment interruptions, treatment discontinuation and regardless of intake of additional medications.

Treatment groups are called early TIPS and standard medical treatment.

*Variable (endpoint):* The endpoint is transplantation-free survival defined as time from randomisation to liver transplantation or death.

*Intercurrent events*: ascites, TIPS implantation and TIPS revision will be ignored.

*Population-level summary:* The population-level summary is defined by the statistical analysis approach.

### Definition of the secondary estimands

#### Estimand 2

Estimand 2 that corresponds to the secondary trial objective to assess the efficacy of TIPS implantation on the course of liver disease is specified by the following 5 attributes. *Population: [A], Treatments: [A].*

*Variable (endpoint):* The endpoint is ascites with need for paracenteses defined as time from randomisation to date of paracentesis.

*Intercurrent events*: transplantation, death will be considered as competing events.

*Population-level summary:* The population-level summary is defined by the statistical analysis approach which is described below.

#### Estimand 3

Estimand 3 that corresponds to the secondary trial objective quality of life is specified by the following 5 attributes.

*Population [B,C]:* 6, 12-month survival of FAS *Treatments:* [A].

*Variable (endpoint):* The endpoint is quality of life measured by SF-36 and CLDQ.

*Intercurrent events:* There are no intercurrent events not already addressed by the specification of population, treatments and variables.

*Population-level summary:* The population-level summary is defined by the statistical analysis approach which is described below.

### Participant timeline {13}

Table 3 summarises the participant timeline including the assessments at each visit.

**Table 3:** Visit schedule and assessments

	**Screening** **(Baseline day)** **−28–0**	**Visit 1** **Rando** **Day 0**	**Visit 2 Intervention** **Day 1–7** after Rando (only TIPS group)	**Visit 3 Discharge 4 days** after intervention** (+ 7/−2 days) **(only TIPS group))	**Visit 4 M1 1 month **after Rando **(+ 2 weeks)**	**Visit 5 M3 3 months **after Rando **(± 4 weeks)**	**Visit 6 M6 6 months **after Rando **(± 4 weeks)**	**Visit 7 M9 9 months **after Rando **(± 4 weeks)**	**Visit 8 M12** **12 months** after Rando **(± 4 weeks)**	FU Every 6 Month until EOS*^2^
Informed Consent	x									
Registration (i.e. entry of first data in eCRF)	x									
Inclusion-/Exclusion criteria	x									
Randomisation		x								
Demographics	x									
Medical history	x									
Physical examination, vital signs	x									
Pregnancy test	x									
12-lead ECG	x									
Colour Doppler ultrasound	x			x	x	x	x	x	x	
Echocardiography (TTE)	x						x			
TIPS (according randomisation)			x							
Laboratory (haematology, clinical chemistry, coagulation)	x**		**:	x	x	x	x	x	x	
Urinalysis	x						x			
Clinical scores^1^	x			x^1^	x^1^	x^1^	x^1^	x^1^	x^1^	
Paracentesis	(x)									
SF-36, CLDQ	x						x		x	
Survival status, liver transplantation				x	x	x	x	x	x	x
Concomitant medication	x	x	x	x	x	x	x	x	x	
(S)AEs	x	x	x	x	x	x	x	x	x	x

*CLDQ *Chronic Liver Disease Questionnaire, *ECG* electrocardiography, *FU* follow up,* Rando* randomisation,* SF-36* Short Form Health Survey (questionnaire)

^*^EOS = end of the entire study is defined as 12 months after randomisation of the last patient

^**^Laboratory tests in screening must be carried out as close as possible to randomisation

(1) Clinical scores include: HE (West Haven Criteria), MELD, MELD Na, MELD 3.0, FIPS, CLIF-C AD, Child-Pugh and NYHA will be documented in eCRF at screening; thereafter, only HE (West Haven Criteria) and NYHA will be collected in eCRF beginning from Visit 3

(2) Only the first paracentesis which is necessary after the study period of 12 months is documented including the date of paracentesis. Further paracenteses after the first one in FU are not considered

### Sample size {14}

Sample size calculation is based on the primary endpoint transplantation-free survival (TFS), defined as time from randomisation to liver transplantation or death. It is assumed that the TFS probability of patients in the control group (SMT) is approximately 66% after one year. With an assumed TFS rate of approximately 82% in the TIPS group, this corresponds to a hazard ratio of 0.48 of the TIPS group compared to the control group (SMT group). The assumption is based on the study by Bureau et al. that analysed 62 patients. The one-year rate of TFS in the TIPS group was 93% (95%CI, 82%–100%) [12]. The TFS probability of the SMT group was assumed according to data from patients with first decompensation with ascites in the INCA trial (unpublished data [24] and, the data provided by Balcar et al. that report1-year TFS in patients with LVP [14]. The effect of TIPS implantation will be assessed by a test at two-sided significance level of 5% and by estimation of the hazard ratio with corresponding asymptotic two-sided 95% confidence interval. The null hypothesis is rejected if the confidence interval does not contain one. Under the above assumptions, the study is planned to detect a difference between the experimental intervention (TIPS group) and the control group (SMT group) with a power of 80%, which requires a total number of 57 events to be observed. The required number of patients to be randomised to observe this amount of events depends on the length of follow-up. With a recruitment period of three years, an additional follow-up period after the end of recruitment of two years, it can be safely assumed that a sufficient number of events will have been observed by the end of the trial if 116 patients are available for analysis (software used, e.g. n Query Advisor 8.3). Furthermore, the exponential drop-out rate is assumed to be 0.01. Therefore, 134 patients will be randomised. Before the end of the planned recruitment phase, a blinded assessment of the TFS rate (both treatments combined) will be performed. If the TFS rate appears to be higher than anticipated, the recruitment phase will be extended, and the required number of patients will be calculated anew under the amended assumptions. Irrespective of this sample size amendment, the analysis will be conducted after the observation of 57 events as planned, so no alpha adjustment is necessary.

### Recruitment {15}

It is intended to randomise 134 patients in approximately 10 centres within 36 months. There is no limit to the number of included patients at an individual study site. Patients with the first single decompensating event with ascites are also often seen in local hospitals and are allocated to a tertiary medical centre mostly only if ascites cannot be controlled after several LVPs and extensive diuretic treatment. In order to fulfil preliminary assumptions on recruitment rates, specific strategies (continuous medical education [CME] events to present the study to local family doctors, out-patient gastroenterologists and local hospitals, providing information material [flyer, presentation]) will be prepared.

## Assignment of interventions: allocation

### Sequence generation {16a}

If the informed consent is obtained, the qualified/authorised personnel will register the patient online in the eCRF (REDCap® system) by creation of a new patient record. The system will automatically assign the next consecutive number (e.g. 1, 2, 3). The unique patient identification number will then consist of a unique centre number assigned by REDCap® combined with the consecutive patient number. The registration date, the date on which the first patient data is entered in the eCRF, will be automatically collected.

The randomisation list will be provided by the biometrician and will be uploaded into REDCap®. The randomisation itself will be performed at the study site on the electronic randomisation form in REDCap®. The randomisation can only be performed if all requirements for randomisation are fulfilled (i.e. eligibility criteria of the patient for randomisation must be confirmed). The randomisation process is started by pushing the “randomise” button in the randomisation form. The randomisation result will be displayed in the form and is saved automatically. A notification of the randomisation result will be sent automatically via E-mail to the responsible data manager of the Clinical Trials Unit (CTU), and the principal investigator at the specific site.

Randomisation will be performed, stratified by site, in blocks of variable length in a ratio of 1:1. The block lengths will be documented separately and will not be disclosed to the sites. The randomisation code will be produced by validated programmes based on the Statistical Analysis System (SAS).

### Concealment mechanism {16b}

The randomisation is done via eCRF (REDCap® system) (see the Sequence generation {16a} section), which permits concealment of the randomisation sequence.

### Implementation {16c}

The randomisation code will be generated by the CTU to ensure that treatment assignment is unbiased and concealed from patients and investigator staff.

## Assignment of interventions: blinding

### Who will be blinded {17a}

Blinding is not possible due to the interventional character of the study.

### Procedure for unblinding if needed {17b}

This is not applicable as the study is designed as an open-label trial.

## Data collection and management

### Plans for assessment and collection of outcomes {18a}

Examinations and assessments will be performed at pre-defined timepoints as specified in Table 2. Data will be entered by the investigator/authorised personnel in the eCRF (REDCap® system). Questionnaires (SF-36 and CLDQ) are first filled out on paper and then transferred to the eCRF. At each visit, patient life status, liver transplantation and paracentesis performed are queried. Events related to TIPS implantation during and after the end of the procedure, categorised incidences for all patients and all other adverse events are collected in a patient safety form in the eCRF.

### Plans to promote participant retention and complete follow-up {18b}

In the first year after randomisation, regular and timely visits support patient contact. After the first year, phone calls also promote long-term follow-up.

### Data management {19}

The setup of the eCRF and data management will be performed with the REDCap® system.

REDCap® uses built-in security features to prevent unauthorised access to patient data, including an encrypted transport protocol for data transmission from the participating sites to the study database. An audit trail provides a history of the data entered, changed, or deleted, indicating the processor and date.

Before any data entry is performed, the trial eCRF will be validated. Site data entry personnel will not be given access to the trial database until they have been trained.

Data will be checked during data entry by programmed warnings and so-called data rules. Furthermore, the Query Module of REDCap® will be used to assign queries manually and control data quality by executing data quality rules. Data corrections will be entered directly into REDCap® by the responsible personnel.

Furthermore, the data will be reviewed for completeness, consistency, plausibility and regarding protocol violations or distinctive medical problems using SAS® software. The resulting queries will be sent to the investigators for correction or verification of the documented data in the eCRF.

### Confidentiality {27}

The investigator must ensure anonymity of the patients; patients must not be identified by names in any documents submitted to the sponsor. Signed informed consent forms and patient enrolment log must be kept strictly confidential to enable patient identification at the site. All study-related information will be stored securely at the study site. All participant information will be stored in locked file cabinets in areas with limited access. All laboratory specimens, reports, data collection, processes and administrative forms will be identified by a coded identification number only to maintain participant confidentiality.

### Plans for collection, laboratory evaluation and storage of biological specimens for genetic or molecular analysis in this trial/future use {33}

Blood and urine samples will be collected at pre-defined timepoints as specified in Table 3. These data are collected in order to assess the safety and efficacy of the therapy in each group. Storage of biospecimens for further research is not planned. Embedded future substudies using data from the eTIPS study must have a specific protocol and must be confirmed by the coordinating investigator. Further, a specific approval from the ethics committee is obtained separately.

## Statistical methods

### Statistical methods for primary and secondary outcomes {20a}

The following populations for analyses are defined: (I) The population targeted by the primary estimand will be called full analysis set (FAS). This means that the patients will be analysed in the treatment arms to which they were randomised. All randomised patients will be included in the FAS. (II) The safety set (SAF) includes all randomised patients who underwent TIPS procedure or received SMT, and patients are analysed according to the received treatment.

The estimand and competing events are described above and summarised in Table 4.

**Table 4:** Estimand and competing events

(Intercurrent) events	Time to	
	**Transplantation free survival**	**Ascites with need for paracenteses**
**Transplantation**	Event	Competing
**Ascites with need for paracenteses**	Ignore	Event
**Death**	Event	Competing
**TIPS implantation**	Ignore	Not available/event
**TIPS revision**	Ignore	Not available/event

The effects of SMT and TIPS implantation with respect to the primary endpoint transplantation-free survival will be estimated and tested by Cox regression. The regression model will include treatment and study site as independent variables, as well as baseline bilirubin, creatinine and age. As an estimate of the effect size, the hazard ratio between the two treatment arms will be given with the corresponding asymptotic two-sided 95% confidence interval. The two-sided test on the difference between SMT and TIPS at significance level 5% will be based on the corresponding asymptotic two-sided 95% confidence interval from the Cox regression model.

The effects of SMT and TIPS implantation with respect to the secondary endpoint time to ascites with need for paracenteses will be estimated and tested by Cox regression. The probability of event over time will be estimated by cumulative incidence rates due to competing events, transplantation and death. The time to event will be compared between the treatment groups with Cox regression models for the event-specific hazard functions using two-sided Wald tests. The regression model will include treatment and study site as independent variables, as well as baseline bilirubin, creatinine and age. As an estimate of the effect size, the event-specific hazard ratio between the two treatment arms will be given with the corresponding asymptotic two-sided 95% confidence interval.

SF-36 and CLDQ will be compared between treatment arms at two different timepoints (months 6, 12). At both timepoints, a mixed linear model for repeated measures (MMRM) is used modelling the respective scale at this and the earlier timepoints, and including randomised treatment and the baseline value of the respective subscale and centre as covariates. Two-sided *p* values of the tests of the hypotheses that the treatment effect is zero at the respective timepoints will be calculated from these models. As estimates of the effect sizes, the differences of adjusted means (TIPS arm vs.SMT arm) will be calculated with two-sided 95% CI.

The total number of AEs, the minimum, maximum and mean number of adverse events (AEs) per patient, the total number of follow-up (FU) days (number of days in the observation period), the number of AEs per FU-day (total number of AEs divided by the total by the number of follow-up days), the number of patients who had at least one AE and the number of patients who stopped treatment due to AE will be given.

The probability of AEs defined by preferred term (PT) according to MedDRA and of AEs defined by system organ class (SOC) according to MedDRA will be estimated by the Aalen-Johansen estimator. For this purpose, the time from randomisation to the first occurrence of the specific type of AE (defined by PT or defined by SOC) will be analysed. For patients not experiencing the specific type of AE, the time to the end of the documentation period or death, whatever occurs first, will enter the analysis and will be treated as a competing event. This will result in probability estimates (cumulative incidences) for the different AE types (defined by PT and by SOC) over time. The results will be displayed in summary tables by showing the cumulative incidences at the maximum observation time. Incidences of AEs will be calculated with 95% confidence intervals. Additionally, the number of non-serious AEs will be summarised by PT and by SOC.

### Interim analyses {21b}

No interim analysis is planned.

### Methods for additional analyses (e.g. subgroup analyses) {20b}

Demographic and other baseline data will be summarised descriptively by a randomised treatment arm. Numbers of complete and missing data (if any) will be shown. Relative frequencies will be shown as valid % (number of patients divided by the number of patients with non-missing values).

### Methods in analysis to handle protocol non-adherence and any statistical methods to handle missing data {20c}

Unless otherwise stated in particular cases, missing values are not replaced and only observed cases are analysed.

### Plans to give access to the full protocol, participant level-data and statistical code {31c}

The sponsor assures that the key design elements of this protocol will be posted in a publicly accessible clinical trials registry.

## Oversight and monitoring

### Composition of the coordinating centre and trial steering committee {5d}

The coordinating centre consists of the principal coordinating investigator (DB, sponsor representative, University Medical Center Freiburg, Germany), the co-principal coordinating investigator (MS), the trial manager (MJ), the statistician (CJ), the data and safety management team (MK and the team from the Clinical Trials Unit Freiburg, Germany) and the monitoring team (HHO, Clinical Trials Unit Freiburg, Germany). The trial steering committee will be represented by the coordinating investigator (DB), co-principal coordinating investigator (MS), head of department of the coordinating centre (RT) and the Data Monitoring Committee (DMC).

### Composition of the data monitoring committee, its role and reporting structure {21a}

An independent Data Monitoring Committee (DMC) is established. The DMC consists of two independent physicians (one interventional radiologist with experience in TIPS implantation and one hepatologists with longterm experience in treatment of patients with portal hypertension) and a statistician. The function of the DMC is to monitor the course of the trial and if necessary to give a recommendation to the coordinating investigator for continuation, modification or discontinuation of the trial. The underlying principles for the DMC are ethical and safety aspects for the patients. It is the task of the DMC to examine whether the conduct of the trial is still ethically justifiable, whether the safety of the patients is ensured, and whether the process of the trial is acceptable. For this, the DMC has to be informed about patient recruitment, adherence to the protocol and observed adverse events. A continuous safety survey of the study will be conducted as part of the annual DMC meetings. The DMC will evaluate mortality, frequency and type of SAEs taking into consideration discontinuation criteria.

### Adverse event reporting and harms {22}

An adverse event (AE) in this study is defined as any untoward medical occurrence/complication in a patient who underwent TIPS or received SMT and which does not necessarily have to have a causal relationship with the TIPS implantation or received therapy.

Table 5 summarises the parameter/adverse events that will be documented to monitor interventional and SMT safety.

**Table 5:** Parameter/adverse events that will be documented to monitor interventional and SMT safety

**During TIPS procedure**	**After the end of the procedure AEs related to TIPS implantation**	**For all patients **
• Injury to carotid artery (perforation) • Procedural pneumothorax • Injury to hepatic artery (perforation/puncture) • Liver capsule perforation • Bile duct injury • Arrhythmia • Allergic reaction • Injury to surrounding organs • Intra-abdominal bleeding • Other	• Complications related to the shunt: – TIPS thrombosis – TIPS dysfunction • Contrast media toxicity • Fistula formation • Other directly related to TIPS procedure	• Bleeding oesophageal varices • Hepatic encephalopathy (HE) beginning from grade II [25] • Spontaneous bacterial peritonitis (SBP) • HRS-AKI • Acute kidney injury (AKI) • Acute on chronic liver failure (ACLF) • Diuretic-induced complications • Cardiac failure (according to NYHA class) • Ischemic hepatitis • Infections • Hepatocellular carcinoma (HCC) (including at least BCLC staging, tumor diameter, vascular invasion etc.) • Other

Adverse events have to be documented in the eCRF starting from the date of ICF signature and until end of the study with the following information: characterisation of the event (listed complication, see above [diagnosis or symptoms, if diagnosis not yet available]), onset/end date, severity according to the current version of CTCAEv5, relationship to procedure (yes/no) or to the shunt (yes/no), serious/non-serious and outcome.

### Frequency and plans for auditing trial conduct {23}

Monitoring is performed by the clinical research associates (CRAs) of the CTU, Medical Center—University of Freiburg. Risk-based monitoring will be conducted according to ICH-GCP E6 and standard operating procedures (SOPs) to verify that patients’ rights and well-being are protected, reported trial data are accurate, complete and verifiable from source documents and that the trial is conducted in compliance with the currently approved protocol/amendment, with ICH-GCP E6 and with the applicable regulatory requirements to ensure patient safety and integrity of clinical trial data.

The investigator will accept monitoring visits before, during and after the clinical trial. Prior to the trial, a site selection visit at each site is conducted to check the prerequisite of the team. Prior to patient recruitment, a site initiation visit at each site is conducted in order to train and introduce the investigators and their staff to the trial protocol, essential documents and related trial-specific procedures, ICH-GCP E6 and national/local regulatory requirements.

On the basis of a risk-based quality management process, the CRA will visit the site regularly depending on trial risk (in this case: low risk) and depending on the recruitment rate and quality of data. During these on-site visits, the CRA verifies that the trial is conducted according to the trial protocol, trial-specific procedures, ICH-GCP, international and national/local regulatory requirements, as applicable. The CRA also performs source data verification and source data review to ensure that the clinical trial data which are recorded in the source data and eCRFs are complete and accurate and to ensure the smooth flow of the processes which are agreed.

In addition to on-site visits, off-site visits can be carried out as long as the centre’s data quality permits (after consulting the project manager and coordinating investigator).

All trial-specific monitoring procedures, monitoring visit frequency and extent of source data verification will be predefined in a trial-specific monitoring plan. The investigator must maintain source documents for each patient in the trial, consisting of case and visit notes (hospital or clinic medical records) containing demographic and medical information, laboratory data, electrocardiograms and the results of any other tests or assessments. All information recorded on eCRFs must be traceable to source documents in the patient’s file. The investigator must also keep the original signed informed consent form (a signed copy is given to the patient).

The investigator must give the CRA direct access to all relevant source documents to confirm their consistency with the eCRF entries and document any protocol violation including corrective and preventive actions taken on a protocol deviation form as soon as possible.

The study may be audited at any time, with appropriate notification, by qualified personnel from the sponsor or an independent external party, to assess compliance with the protocol, good clinical practice (GCP) and regulatory requirements. The study may also be inspected by health authority inspectors, after appropriate notification. The investigator needs to inform the CTU immediately of an inspection requested by a regulatory authority. In the event of an audit or an inspection, the investigator should ensure that direct access to all study documentation/data, including source documents, will be granted to the auditors or inspectors.

### Plans for communicating important protocol amendments to relevant parties (e.g. trial participants, ethical committees) {25}

Any change or addition to the protocol can only be made in a written protocol amendment that must be approved by the sponsor, competent authority where required, and the independent ethics committee (IEC). Only changes to the protocol that are required for patient safety may be implemented prior to IEC approval. Regardless of the need for approval of formal protocol amendments, the investigator is expected to take immediate action required for the safety of any patient included in this trial, even if this action represents a deviation from the protocol. In such cases, the sponsor has to be notified as soon as possible of this action; the IEC should be informed correspondingly. Information regarding important protocol modifications will be provided in due time to further relevant parties (e.g. investigators, trial participants, trial registries, journals).

#### Dissemination plans {31a}

Upon trial completion, the results of this trial will be submitted for publication and/or posted in a publicly accessible database of clinical trial results irrespective of the results of the trial. Reporting guidelines will be taken into account (see www.equator-network.org), e.g. the CONSORT will be adhered to in the preparation of papers on the results of randomised studies. Each publication of trial results will be in mutual agreement between the principal investigator, the other investigators involved and the CTU. All data collected in connection with the clinical trial will be treated in confidence by the coordinating investigator and all others involved in the trial until publication. Interim data and final results may only be published (orally or in writing) with the agreement of the coordinating investigator and the CTU. For authorship of the final clinical trial report and in publications of the trial protocol and results, the authorship criteria for manuscripts submitted for publication defined by the International Committee of Medical Journal Editors will be adhered to.

## Discussion

TIPS implantation has emerged as an important interventional treatment of complications of portal hypertension. Patients with liver cirrhosis and refractory ascites who are allocated to TIPS implantation are at risk for failure of ascites control [13]. One possible reason is that TIPS implantation is performed too late in these patients and that compensatory mechanisms may persist despite successful TIPS implantation [12, 13]. In the study by Bureau et al., patients were allocated to TIPS implantation after a mean of 4.5 paracenteses indicating that these patients did not fulfil the definition of refractory ascites. Interestingly, these patients showed excellent 1-year transplantation-free survival. Further, it has been shown that a lower paracentesis frequency before TIPS is associated with better ascites clearance after TIPS implantation [12]. Previously, a small retrospective study (*n* = 27) was published analysing the effect of anticipant TIPS implantation in patients with the first episode of ascites or variceal bleeding. Liver-related events were significantly reduced and overall survival was better in patients with anticipant TIPS implantation [26]. However, this study may harbour significant bias due to its retrospective design. One major issue is that patient selection for anticipant TIPS implantation was not predefined. Balcar et al. provide some evidence that patients with first decompensation of grade 2 ascites and a MELD score ≥ 15 as well as patients with grade 3 ascites irrespective of the MELD score may benefit from early TIPS implantation as these patients have a high risk of further decompensation and consecutively a higher risk for death [14]. As to date, there are no prospective data showing that TIPS implantation is associated with improved transplantation-free survival in these selected patients with first ascitic decompensation; our study aims to close an important gap in patients with ascites and may have the potential to change treatment algorithms.

### Trial status

The recruitment will start in March 2025, and the recruitment process is planned to be completed by March 2028. This is study protocol version 1.3, 03.07.2025.

## Data Availability

The current version of the protocol is registered at ClinicalTrials.gov with the following ID: NCT06576934 and in the Deutsches Register Klinische Studien (DRKS): DRKS00034545. It is planned to publish the results of the trial in a peer-reviewed journal. Additionally, the final trial dataset will be provided upon request.

## Abbreviations

ACLF: Acute-on-chronic liver failure
AE: Adverse event
AKI: Acute kidney injury
CME: Continuous medical education
CTU: Clinical Trials Unit
DMC: Data Monitoring Committee
EASL: European Association for the Study of the Liver
eCRF: Electronic case report form
ePTFE: Expansion polytetrafluoroethylene
FAS: Full analysis set
GCP: Good clinical practice
HBV: Hepatitis B virus
HCV: Hepatitis C virus
HE: Hepatic encephalopathy
HVPG: Hepatic venous pressure gradient
IEC: Independent ethics committee
LVP: Large-volume paracenteses
MELD: Model of end-stage liver disease
MMRM: Mixed linear model for repeated measures
NSBB: Non-selective beta-blocker
NYHA: New York Heart Association
PSG: Portosystemic gradient
IVC: Inferior vena cava
PT: Preferred term
PVT: Portal vein thrombosis
QOL: Quality of life
CRA: Clinical research associate
SAF: Safety set
SAS: Statistical Analysis System
SBP: Spontaneous bacterial peritonitis
SMT: Standard medical treatment
SOC: System organ class
SOP: Standard operating procedure
TFS: Transplantation-free survival
TIPS: Transjugular intrahepatic portosystemic shunt
TTE: Transthoracic echocardiogram
